# The level of provision of specialist palliative care services in Scotland: an international benchmarking study

**DOI:** 10.1136/bmjspcare-2016-001301

**Published:** 2017-07-08

**Authors:** Hamilton Inbadas, José Miguel Carrasco, Michelle Gillies, David Clark

**Affiliations:** 1 University of Glasgow, School of Interdisciplinary Studies, Dumfries, UK; 2 University of Navarra, ATLANTES Research Programme, Institute for Culture and Society, Navarra Institute for Health Research, Pamplona, Spain; 3 Scottish Public Health Network, NHS Health Scotland, Scotland

**Keywords:** specialist palliative care, service provision, coverage, EU comparisons, scotland

## Abstract

**Objectives:**

Comparative benchmarking of specialist palliative care (SPC) services across jurisdictions can be used to assess the adequacy of provision. Published in 2016, the *Scottish Atlas of Palliative Care* unlocks the possibility of benchmarking Scotland’s provision against other European Union (EU) countries. Our objectives were to describe the provision of SPC services in Scotland and compare this with other EU countries, assessing coverage against European norms.

**Methods:**

We conducted a secondary analysis of data collected as part for the *Scottish Atlas* by structured telephone (n=33) or online (n=3) survey with informants from 14 territorial health boards and 15 hospices who provided information about SPC services in their locality. National-level Scottish data were compared with data from other EU countries allowing ranking for each service type and service coverage as calculated against European Association for Palliative Care norms.

**Results:**

Scotland had a total of 23 SPC inpatient units containing 349 beds, 27 SPC hospital support teams and 38 SPC home care teams. Relative to other EU countries, Scotland ranked seventh for provision of SPC inpatient units and hospital support teams, and fifth for home care teams. Coverage for these services was 85%, 100% and 72%, respectively.

**Conclusion:**

Scotland is positioned among the top 10 EU countries for the level of provision of SPC services. National policy in Scotland has focused on the delivery of palliative care at home or in a homely setting. These data support a focus on developing services in community settings to meet Scotland’s policy ambitions.

## Background

It is well established that decision making about the development of specialist palliative care (SPC) services, if it is to meet the needs, preferences and priorities of local communities, should be underpinned by accurate and current information characterising existing service provision. This includes the type of services available, their geographic range and their organisational capacity. A 2015 report by the Scottish Parliament identified a significant ‘information deficit’ concerning the provision of SPC in Scotland.^1^ In response, a *Scottish Atlas of Palliative Care* was produced in 2016, which described, at national and territorial health board levels, the provision of SPC services in Scotland.^2^

While international benchmarking studies^3 4^ have described the development and organisation of SPC services across the whole WHO European Region as well as the countries of the European Union (EU), it has not been possible to profile Scotland in these, since they include the UK as a whole, and not its constituent countries, as the unit of analysis. The data required to profile Scotland against the UK as well as other EU member states using ranking and coverage models was collected for the first time in the *Scottish Atlas*. We now use the primary data collected for the *Scottish Atlas* to establish the level of SPC provision in Scotland as compared with other EU countries and to express this as a measure of coverage for specific service types based on expert guidelines.^5 6^

## Aims

The aims of this study were to describe, at national level, the provision of SPC services in Scotland, to compare these with other countries in the EU and to assess the coverage of SPC services in Scotland based on the recommendations of a European Association for Palliative Care (EAPC) ‘White Paper’ on standards and norms for hospice and palliative care in Europe.

## Methods

In Scotland, healthcare is provided free at the point of access to all citizens through the National Health Service (NHS), which is organised into 14 territorial health boards. SPC services are also provided by independent hospices, which are charitable organisations that receive up to 50% of their agreed annual operating costs from the NHS. We conducted a secondary analysis of aggregated national level data on the level of SPC service provision that had been collected in a national service mapping study as part of the *Scottish Atlas of Palliative Care*.^2^ The methods used to map SPC service provision in the *Scottish Atlas* have been described previously.^2^ In brief, the chief executive officers of independent hospices, NHS Executive Leads for Palliative and End of Life Care and, where in place, the chairperson of the Managed Clinical Network for Palliative and End of Life Care in each territorial health board were contacted between August and September 2015, informed of the nature and purpose of the service mapping study and invited to contribute information about the services they provided. Consent was implied by the willingness of service providers to nominate a key informant from their organisation to contribute data to the study. Data were collected in a telephone survey conducted by one of three trained investigators (HI, MG and AJW). Prior to the survey, respondents were sent a structured questionnaire to enable them to gather the required information in advance. All survey respondents were either directly involved in delivering or responsible for managing the SPC services on which they provided information; in some cases, it was necessary to speak with several informants from a single territorial health board to obtain a complete picture of service provision. Where it was not possible to conduct a telephone survey, despite repeated attempts, respondents were given an option to complete the structured questionnaire online (n=3). The structured questionnaire and definitions therein were derived from the *European Atlas of Palliative Care* 7 and adapted for local use (see appendix 1 of the *Atlas*). In total, 36 informants provided data from all 14 territorial health boards and all 15 fully operational independent hospices. The SPC service mapping study did not involve patients or service users. It is anomalous that information of this nature is not routinely available to inform the design and delivery of SPC services to meet the needs of local populations. The service mapping study was therefore considered as service evaluation and, as such, research ethics approval for the study was not sought.

We followed the method of measuring palliative care ‘resources’ developed in previous studies.^3^ This involved establishing the numbers of three types of SPC service provision, inpatient units, hospital support teams and home care teams, then calculating the ratio of services per million inhabitants by service type and total number of all services. This ratio was used to compare Scotland with the top ranking countries in the EU.^3^ Population data were obtained from National Records Scotland.^8^

Following an established method, we also calculated coverage of SPC provision in Scotland for each service type.^4^ Coverage refers to the relationship between the number of available services and the estimated number of services that would be required, based on expert opinion, to meet the palliative care needs of a given population. The EAPC White Paper identifies ‘norms’ for the structural provision of SPC, which represent a consensus statement of the levels that, if achieved, would be expected to translate into high-quality care. Recommendations based on these ‘norms’ for the number of services required per population are as follows: one home care team per 100 000 inhabitants; one inpatient unit and one hospital support team per 200 000 inhabitants.^5 6^ Based on these figures, the required number of the three types of services was calculated for the population of Scotland and a measure of ‘coverage’, expressed as a percentage, was established. EAPC recommendation concerning the desirable upper and lower numbers of SPC beds per million population, producing a calculation based on the number of SPC inpatient beds in Scotland, was also calculated.^5^

## Results

### Level of SPC service provision

Scotland had a total of 23 SPC inpatient units containing 349 beds, 27 hospital support teams and 38 home care teams, which equates to 4.3 SPC inpatient units per million people, 5.0 hospital support teams per million people and 7.1 home care teams per million people. Scotland’s position for each type of service in relation to the 10 top EU countries is shown in table 1.

**Table 1:** Comparison of types of specialist palliative care in Scotland and other European countries

	Inpatient units*		Hospital support teams†		Home care teams‡	
	Country	Ratio/million	Country	Ratio/million	Country	Ratio/million
**1**	The Netherlands	12.7	Belgium	10.4	Estonia	11.3
**2**	Luxembourg	9.4	Ireland	8.5	Sweden	11.2
**3**	Germany	5.2	Slovenia	8.3	Poland	8.4
**4**	Denmark	5.0	UK	5.7	Ireland	7.6
**5**	Belgium	4.6	Luxembourg	5.7	Scotland	7.1
**6**	Austria	4.4	France	5.3	Hungary	7.0
**7**	Scotland	4.3	Scotland	5.0	UK	6.1
**8**	Sweden	4.0	Malta	3.6	Austria	5.8
**9**	Poland	3.8	Latvia	3.4	Luxembourg	5.7
**10**	UK	3.5	Austria	3.4	Italy	5.2

*Scotland has 23 palliative care inpatient units containing 349 beds.

†Scotland has 27 hospital support teams.

‡Scotland has 38 home care teams.

### Coverage

Applying the EAPC norms to Scotland’s population of 5 347 600,^8^ the optimal level of provision for each SPC service is 27 inpatient units, 27 hospital support teams and 53 home care teams. Based on the number of services available in Scotland, we estimate coverage of SPC inpatient units, hospital support teams and home care teams to be 85%, 100% and 72%, respectively. This positions Scotland among the top 10 EU countries for coverage in all three types of service provision (table 2).

**Table 2:** Coverage of specialist palliative care in Scotland based on EAPC White Paper recommendations (top 10 EU countries)

	Inpatient units		Hospital support teams		Home care teams	
	Country	Coverage (%)	Country	Coverage (%)	Country	Coverage (%)
**1**	The Netherlands	254	Belgium	215	Sweden	113
**2**	Luxembourg	153	Ireland	170	Estonia	112
**3**	Germany	102	Slovenia	167	Poland	84
**4**	Denmark	100	UK	115	Ireland	76
**5**	Belgium	95	Luxembourg	100	Scotland	72
**6**	Austria	88	Scotland	100	Hungary	69
**7**	Scotland	85	France	82	UK	62
**8**	Sweden	80	Malta	72	Austria	58
**9**	Poland	76	Austria	69	Luxembourg	57
**10**	United Kingdom	70	Latvia	63	Italy	51

EAPC, European Association for Palliative Care.' at the bottom of Tables 2 and 3.

The EAPC norms recommend a range of between 80 and 100 SPC inpatient beds per million people. Applied to Scotland’s population, this would equate to a recommended number of inpatient beds ranging from 427 to 535. Although relative to the EAPC norms, the coverage of SPC inpatient units is 85% in Scotland, the coverage of SPC inpatient beds is 65% (at 100 per million) and 82% (at 80 per million) (table 3).

**Table 3:** Comparison of number of SPC inpatient units and inpatient beds in Scotland against EAPC norms

	Recommended	Available	Coverage (%)
Inpatient units*	27	23	85
At 80 *beds* per million†	428	349	82
At 100 *beds* per million†	535	349	65

*4.3 units per million inhabitants is based on 5 PC units (50 beds) per million

†EAPC White Paper upgraded recommendation

## Discussion

To date, published studies examining the provision of SPC services at a regional, national or international level have largely relied on information provided by one or more national experts.^3 9 10^ This is problematic because national experts may lack the requisite knowledge to accurately report in detail service provision at the national or subnational level and may be biased in their reporting.^11^ Our study analysed data gathered from 36 informants directly delivering, or responsible for managing, local SPC services in each of Scotland’s 14 territorial health boards areas, increasing the validity of our findings. Due to their local knowledge and the nature of the telephone survey, most respondents volunteered a narrative around the quantitative data on the level of service provision they provided, which was invaluable in contextualising the study findings. To our knowledge, this is the first study of its kind to use such a robust data collection strategy.

The UK is generally considered to be the reference country against which the development of SPC in other countries is benchmarked.^1^ Until now, it has not been possible to disaggregate Scottish data from that of the rest of the UK.^3 9 10^ While we might expect the provision of SPC services in Scotland to be similar to the rest of the UK, this is the first study to provide confirmation. The provision of SPC services in Scotland was found to be well balanced and broadly reflective of that in the rest of the UK, with Scotland’s position being higher than the rest of the UK for inpatient and home care teams but lower for hospital support team. Analysis of coverage against EAPC norms suggests that Scotland has optimal coverage for hospital support teams. More broadly, Scotland was positioned among the top 10 EU countries in hospital and home care resources for SPC. Although the ratio of home care teams per million people (7.1) is better than the ratios for hospital support teams (5.0) and inpatient units (4.3), Scotland shows an optimal coverage of hospital support teams and a lower than recommended coverage for inpatients units (85%) and home care teams (72%). Calculated at both the upper and lower ranges, Scotland scores lower on SPC inpatient bed coverage (65%–82%) than it does on the calculation based on the number of units.

For almost two decades, the strategic narrative for health and social care in Scotland has focused on the delivery of safe, effective, high-quality, patient-centred care delivered at home or in a homely setting.^12 13^ As a result, over the last decade, the total number of available staffed hospital beds in Scotland has been reduced by almost a fifth.^14^ The lower than anticipated level of inpatient SPC beds may therefore reflect successful policy implementation rather than deficiencies in the planning and provision of SPC services. Many service providers in our study, especially in remote and rural areas, identified that while they did not have dedicated SPC inpatient beds, they were providing integrated SPC to inpatients that remained under the care of their admitting specialty, through SPC hospital support teams. This flexible model of care ensures effective use of acute hospital beds and continuity of care and enables collaborative working between generalists and specialists that in turn may facilitate the ‘up-skilling’ of healthcare providers from other specialties in delivering ‘generalist’ palliative care. Indeed, in recent years, the role of SPC providers in providing education and support to generalists has been championed.^15 16^ Increasingly in Scotland SPC clinicians and nurse specialists are jointly appointed to work between the independent hospices, the acute hospital sector and community services to provide integrated care and facilitate early hospital discharge. This change in how and where SPC teams are based is reflected in the optimal coverage of hospital support teams.

The coverage of home care teams in Scotland was less than optimal. This is an important finding. If the policy of shifting the balance of care from hospitals to community settings is to be translated into practice, community services must be adequately resourced.^12^ There has been a growing policy recognition in Scotland of the importance of achieving preferred place of care and preferred place of death.^14^ Most people would prefer to die at home or in a community setting.^17^ It is not possible to measure how well preferences are being met in Scotland because neither preferred place of care and preferred place of death are systematically recorded. In England, an indicator for death in usual place of residence has been developed and is in use. In Scotland, such a measure is not available. A surrogate measure, the percentage of the last 6 months of life spent at home or in a community setting, is in use; in 2014/2015 among decedents in Scotland, 86% of the last 6 months of life were spent at home or in a community setting and 14% in an acute hospital.^18^ Moreover, people are more likely to die in hospital in Scotland (53%) than they are in England (47%).^19^ Adequately resourcing community-based services, across health and social care, will be important if Scotland is to realise its policy ambitions; our study provides data to advocate for further developing community-based services. Many other health and social care professionals contribute to the interdisciplinary teams that support people to live and die well in community settings, not least community district nurses, disease-specific specialist nurses and primary care physicians. In Scotland, these key individuals provide generalist palliative care and often act as gatekeepers to SPC services. The availability of high-quality generalist palliative care is likely to directly impact on the demand for and subsequently delivery of SPC services in community settings. At the same time, measuring the availability and delivery of generalist palliative care raised additional challenges. It is important to note that when collecting the primary data for the *Atlas*, despite clear definitions, there was variation in how service providers conceptualised their services; for example, one territorial health board reported having just one home care team while another, with the same level of staffing across its home care service, reported having multiple home care teams stating that each team covered distinct geographical areas within the board. Our data on home care teams should therefore be interpreted with caution. This does raise questions about what ‘counts’ as a service in any given setting and may have led to an underestimation of the number of home care teams operating in Scotland. It also highlights the importance of common definitions and the challenges in operationalising these, both for service evaluation and for research.

The EAPC White Paper provides norms for the number of services and resources per million people, allowing for the diversity of concepts of SPC and varying cultures across Europe. These norms are based on expert opinion and estimates of demand for services that EAPC considers, if achieved, will translate into the provision of high-quality care. This approach to establishing a range of values for benchmarking is not without limitations. It is common practice where the evidence base is lacking to rely on expert opinion. Underpinning recommendations for service provision using estimated demand for services, rather than need, is problematic; however, estimating palliative care needs is particularly challenging.^20^ The appropriateness of these measures is likely to change as services evolve and the evidence base grows. The measures may also have differing significance according to the context of the healthcare system within a country. For example, the coverage of hospital support teams in Belgium was over 100%; however, according to Belgian law, every hospital is obliged to have a SPC team. The extent to which ‘generalist’ palliative care principles are effectively embedded within a healthcare system may also influence the required level of SPC services and in part explain an apparent underprovision or overprovision of certain services. It is important to consider that these metrics are not sensitive enough to capture such nuance. The balance between services could also be a result of the distribution of population across different areas of the country. With the exception of the central belt of Scotland, where the population is concentrated into large conurbations, there are many dispersed and rural communities of small population. As in many other countries, the distribution and volume of SPC services in Scotland is the result of historical circumstances, local priorities and enthusiasms, as well as the ebb and flow of government interest and funding. The EAPC norms provide a valuable tool for benchmarking the provision of SPC services between countries or within a country over time, which can be a valuable tool for advocacy and also, over time, for more rational planning. However, these measure require careful interpretation considering the wider sociodemographic, political and economic context within which services are being provided. Importantly, these metrics do not provide any objective measure of the quality of SPC.

The difference in timing of data gathered for Scotland alone and that gathered for the 10 EU countries used as comparators is acknowledged. Data for the EU ranking and coverage studies were collected in 2013, compared with 2015 for the present study. This small time gap is not considered to be significant. Indeed, the proximity of Scottish data to that for the UK as a whole, given the common evolution of SPC provision across the UK, is reassuring.

The primary data used in this study were derived from a novel SPC service mapping study. Prior to this, it was not possible to describe the level of SPC provision at local or national level in Scotland; it is still not possible to measure SPC activity, outcomes or quality of care in Scotland. It is anomalous that data of this kind are not available to inform the design, strategic commissioning, delivery and continuous quality improvement of SPC services. These significant ‘information deficits’ were recognised in a 2015 Scottish Parliamentary Inquiry into Palliative Care,^1^ and a policy commitment has been made to address them in Scotland’s Strategic Framework for Action on Palliative and End of Life Care later that year by the Scottish Government.^15^ The national mapping survey was a first step towards this. The study was both labour and time intensive; however, it was necessary to provide reliable information to quantify and characterise SPC provision at local and national level in Scotland. In the study, several informants acknowledged that their service(s) were being redesigned to better meet the needs, preferences and priorities for care in their locality, as informed by local health needs assessments. This highlights the challenge of maintaining accurate and current data on SPC service provision and organisation at a national level. A more sustainable approach to collecting and collating these data is required building on models of SPC directories used elsewhere and making use of multiple providers of data.

Finally, we acknowledge that this is an analysis of indicators for SPC. It does not address the levels and coverage of resources that may be available for the delivery of ‘generalist’ or ‘primary’ palliative care. Such a mapping and assessment is also worth considering but was beyond the remit of the present study.

## Conclusion

This study has addressed part of the ‘information deficit’ previously identified. It provides a comparative analysis of resources and coverage of SPC in Scotland, as compared with other countries of the EU. In all the items compared - inpatient units, hospital care support teams and home care teams — Scotland is positioned in the top 10 EU countries and has a consistent level of coverage. Moreover, this is at a high level (72%–100%) in relation to the expert recommendations of the EAPC.

The study provides a baseline from which to monitor the development of SPC in Scotland. The Scottish government has published a *Strategic Framework for Action for Palliative and End of Life Care* covering the period 2016–2021.^15^ The present study and the accompanying *Atlas* constitute an important baseline, against which some of the goals of the *Framework* can be assessed.

## References

[1] Health and Sport Committee. We need to talk about palliative care. secondary we need to talk about palliative care. 2015 http://www.parliament.scot/S4_HealthandSportCommittee/Reports/HSS042015R15.pdf

[2] Inbadas H, Gillies M, Clark D Scottish atlas of Palliative Care. Glasgow: University of Glasgow, 2016.

[3] Woitha K, Garralda E, Martin-Moreno JM, et al Ranking of Palliative Care Development in the Countries of the European Union. J Pain Symptom Manage 2016;52:370–7. 10.1016/j.jpainsymman.2016.03.008 27287622

[4] Centeno C, Lynch T, Garralda E, et al Coverage and development of specialist palliative care services across the World Health Organization European Region (2005-2012): Results from a European Association for Palliative Care Task Force survey of 53 countries. Palliat Med 2016;30:351–62. 10.1177/0269216315598671 26231421 PMC4800456

[5] Radbruch L, Payne S White paper on standards and norms for hospice and palliative care in Europe: part 1. European journal of palliative care 2009;16:278–89.

[6] Radbruch L, Payne S White Paper on standards and norms for hospice and palliative care in Europe: part 2. European Journal of Palliative Care 2010;17:22–33.

[7] Centeno-Cortes C, Lynch T, Donea O, et al EAPC atlas of palliative care in Europe 2013 - full edition. Milano: European Association for Palliative Care, 2013.

[8] National Records of Scotland. Population, 2014 http://www.nrscotland.gov.uk/statistics-and-data/statistics/statistics-by-theme/population

[9] Economist Intelligence Unit. The 2015 Quality of Death Index: ranking palliative care across the world. London: The Economist Intelligence Unit, 2015.

[10] Lynch T, Connor S, Clark D Mapping levels of palliative care development: a global update. J Pain Symptom Manage 2013;45:1094–106. 10.1016/j.jpainsymman.2012.05.011 23017628

[11] Loucka M, Payne S, Brearley S EURO IMPACT. How to measure the international development of palliative care? A critique and discussion of current approaches. J Pain Symptom Manage 2014;47:154–65. 10.1016/j.jpainsymman.2013.02.013 23770077

[12] The Scottish Government. Better Health, Better Care: action Plan. Edinburgh: The Scottish Government, 2007.

[13] The Scottish Government. 2020 Vision. 2011 http://www.gov.scot/Topics/Health/Policy/2020-Vision

[14] Information Services Division. Hospital care: beds, 2010 http://www.isdscotland.org/Health-Topics/Hospital-Care/Beds/

[15] The Scottish Government. Strategic Framework for Action for Palliative and End of Life Care, 2015 http://www.gov.scot/Resource/0049/00491388.pdf

[16] The Scottish Government. Government TS, Living and Dying Well: a national action plan for palliative and end of life care in Scotland. Edinburgh: The Scottish Government, 2008.

[17] Ryder S A time and a place what people want at the end of life, 2013 http://www.sueryder.org/about-us/policies-and-campaigns/our-campaigns/dying-isnt-working/~/media/files/about-us/a-time-and-a-place-sue-ryder.ashx

[18] Information Services Division. Health and Social Care: data Tables. Data Tables: Secondary Health and Social Care, 2016 http://www.isdscotland.org/Health-Topics/Health-and-Social-Community-Care/Publications/data-tables.asp?id=1721#1721

[19] Public Health England. End of Life Care Profiles. Secondary End of Life Care Profiles. http://fingertips.phe.org.uk/profile/end-of-life/data#page/0/gid/1938132883/pat/6/par/E12000004/ati/102/are/E06000015

[20] Murtagh FE, Bausewein C, Verne J, et al How many people need palliative care? A study developing and comparing methods for population-based estimates. Palliat Med 2014;28:49–58. 10.1177/0269216313489367 23695827

